# Autologous vascular proximal fibular graft in the treatment of giant cell tumor of the distal radius—a case report

**DOI:** 10.1093/jscr/rjae490

**Published:** 2024-08-08

**Authors:** Teodora Todorova, Nikola Gramatnikovski, Tamara Angelovska, Slavica Kostadinova-Kunovska, Nevena Manevska, Marta Foteva, Milan Samardziski

**Affiliations:** Department of Orthopedic Oncology, University Clinic for Orthopedic Surgery, 1000 Skopje, Republic of North Macedonia; University Clinic for Thoracic and Vascular Surgery, 1000 Skopje, Republic of North Macedonia; Institute of Pathology, Faculty of Medicine, Ss, Cyril and Methodius University, 1000 Skopje, Republic of North Macedonia; Institute of Pathology, Faculty of Medicine, Ss, Cyril and Methodius University, 1000 Skopje, Republic of North Macedonia; Institute of Pathophysiology and Nuclear Medicine, Faculty of Medicine, Ss, Cyril and Methodius University, 1000 Skopje, Republic of North Macedonia; Department of Hand Surgery, University Clinic for Orthopedic Surgery, 1000 Skopje, Republic of North Macedonia; Department of Orthopedic Oncology, University Clinic for Orthopedic Surgery, 1000 Skopje, Republic of North Macedonia

**Keywords:** autologous proximal fibular vascular graft, bone scan, distal radius

## Abstract

Giant cell tumor of bone (GCTB) represents an intermediate, locally aggressive tumor, with a peak of incidence in the third decade of life with female predominance (2:1). The distal radius is the third most common localization and especially challenging in the treatment is saving the wrist joint function. In this report, we present a case of a 32-year-old patient diagnosed with a giant cell tumor of the distal radius, primarily treated with curettage of the bone. Due to aggressive tumor recurrence, considering local control of the tumor, we decided to perform a resection of the distal radius and reconstruction with an autologous proximal vascular fibular graft. We performed a SPECT/CT scan to confirm the functionality of the graft. We find this procedure a safe technique for local control of tumor recurrence and an ideal substitute for a limb salvage procedure.

## Introduction

Since its first description in 1818 by Cooper, giant cell tumor of bone (GCTB) has remained a therapeutic challenge for orthopedic surgeons [1, 2].

This is mostly due to its high local recurrence rate, potential joint functional impairment, and possible metastases.

Different surgical treatment modalities come into consideration, and for each of them, various functional results have been reported in the studies available. They have evolved from amputation to limb salvage surgery, and they can vary from curettage to wide resection depending on the tumor [3].

## Case report

We present a case of a 32-year-old female patient who presented with pain and swelling in the area of the right wrist. On the initial X-ray, an osteolytic lesion of the distal part of the right radius was detected, highly suspicious for GCTB. Curettage of the lesion was performed and the histological examination of the specimen confirmed the previously suspected diagnosis.

**Figure 1:**
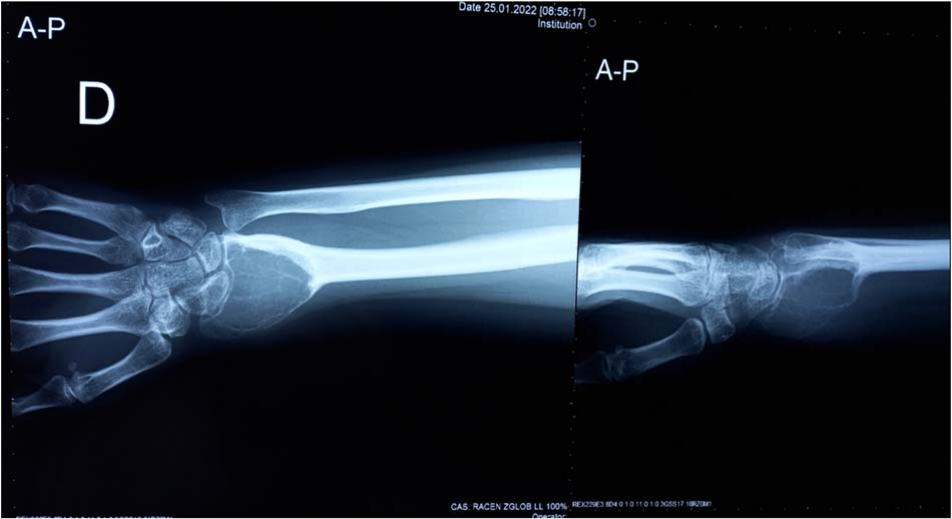
Plane anterior–posterior X-ray of the right forearm.

Three years after the first surgery, the patient experienced pain and swelling in the area of the right wrist, highly suspicious of local tumor recurrence (Fig. 1). Due to aggressive local recurrence, in order to achieve local control of the lesion, and after Tumor Board discussion, we decided to perform wide resection of the distal radius and reconstruction with autologous vascularized fibular graft with harvestation of the inferior lateral geniculate branch and peroneal vessels.

**Figure 2:**
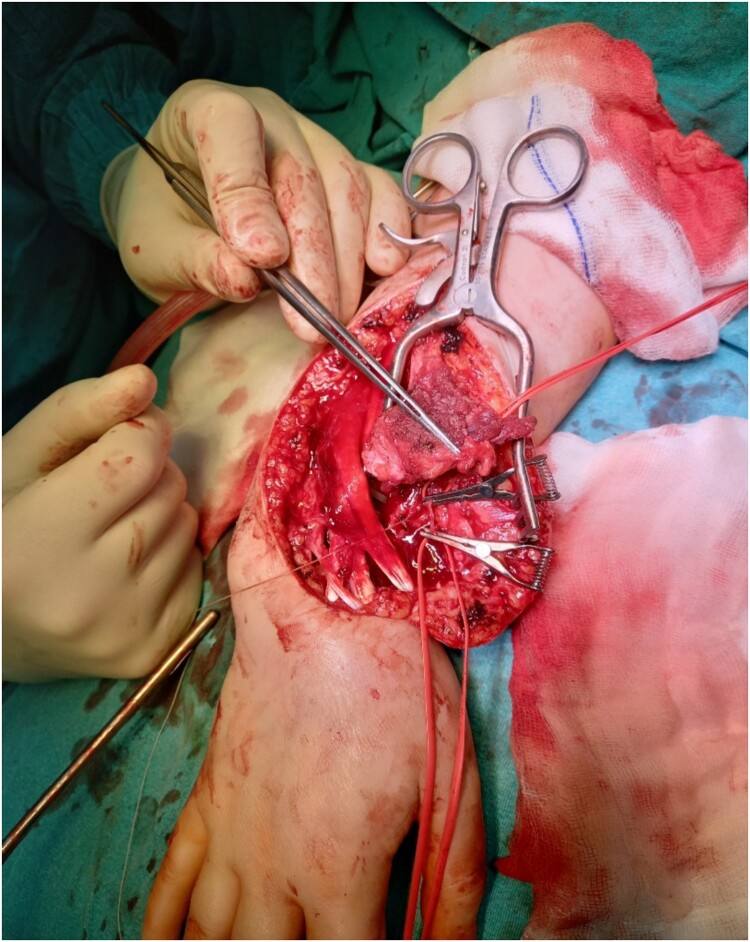
Reconstruction of the defect after resection of the GCTB of the distal radius and anastomosis of the vessels.

**Figure 3:**
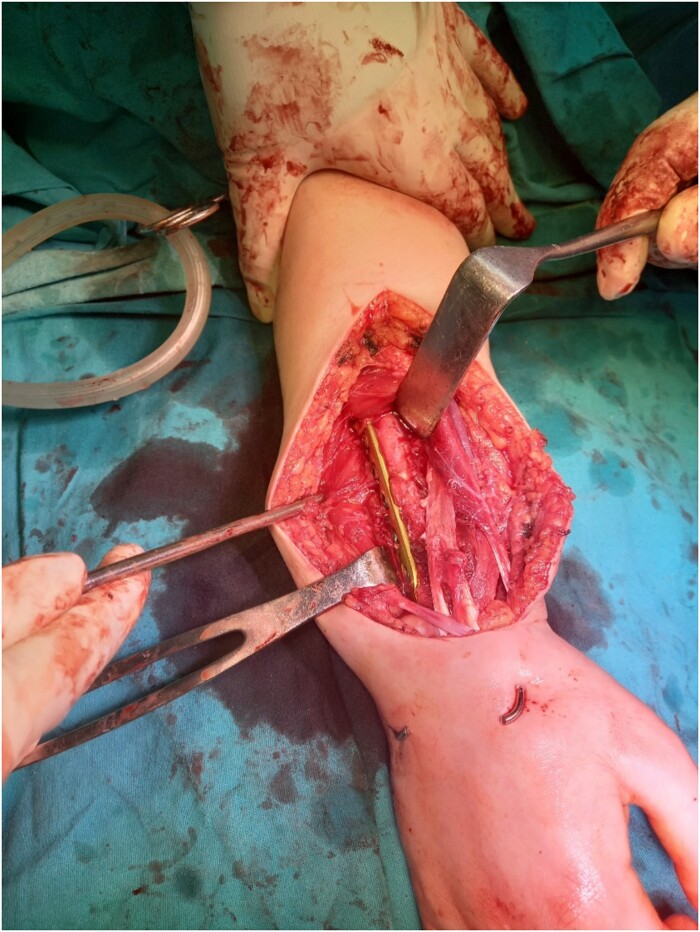
Graft fixation with plate and screws, and two Kirschner wires.

Resection of the tumor and harvesting of the bone graft from the ipsilateral proximal fibula was done simultaneously. We harvested a graft with a length of 8 cm with an inferior lateral geniculate branch and peroneal vessels to be used in the reconstruction of the defect that remained after the resection of the tumor (Fig. 2). After placing the graft in the place of the defect, the inferior lateral geniculate branch was anastomosed through a terminal-lateral anastomosis with the radial artery, while the vein was connected to the cephalic vein through an end-to-end anastomosis. The graft was fixed with a plate and screws, and two Kirschner wires (Fig. 3).

**Figure 4:**
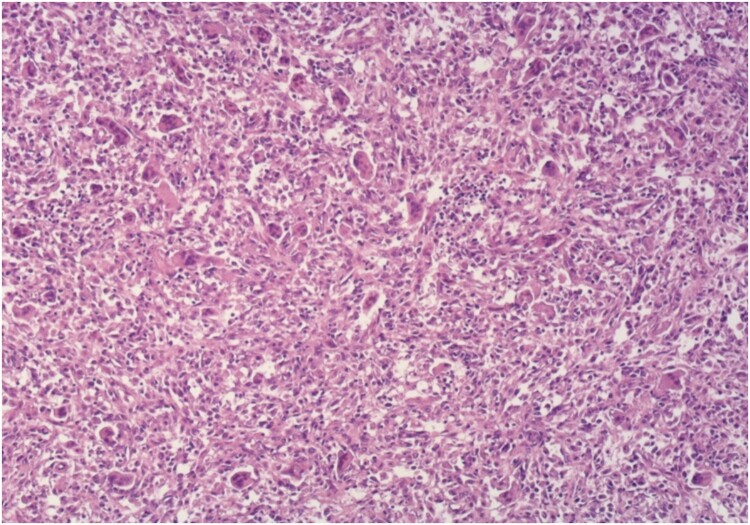
Microscopic appearance of the tumor, Ne-Eo, ×40.

Histology confirmed a recurrence of GCT, composed of numerous multi-nucleated giant cells in-between round to oval mononuclear cells with identical nuclear morphology, with focal collagen deposition, as well as wide areas of recent and older hemorrhage (Fig. 4).

**Figure 5:**
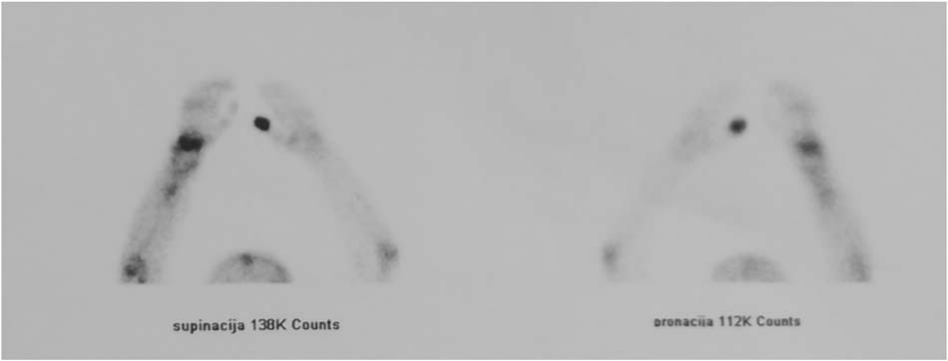
Bone scan confirmed the functionality of the graft.

**Figure 6:**
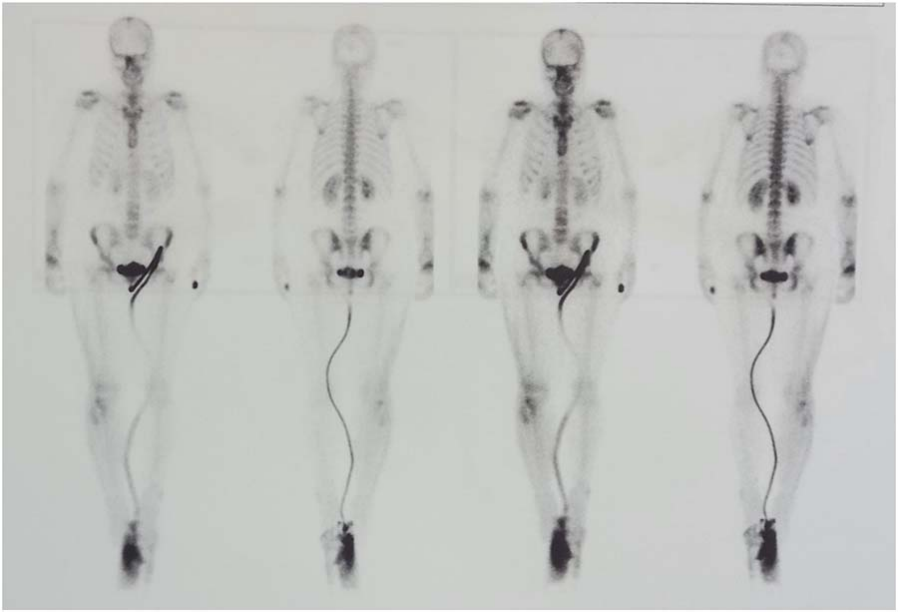
Bone scan confirmed the functionality of the graft.

We performed two bone scans, one before and one after the surgical intervention, using 99mTc-MDP as a bone-seeking agent. A preoperative bone scan revealed increased pathologic accumulation of the bone-seeking radiotracer on the late static images in the right distal radius, conclusive with the localization of the GTC. The postoperative bone scan with SPECT/CT study, one month after the surgical procedure, detected diffuse emphasized blood pool phase as well as an increased accumulation of the tracer in the right distal radius, which corresponded to the viable vascular graft implanted (Figs 5 and 6).

**Figure 7:**
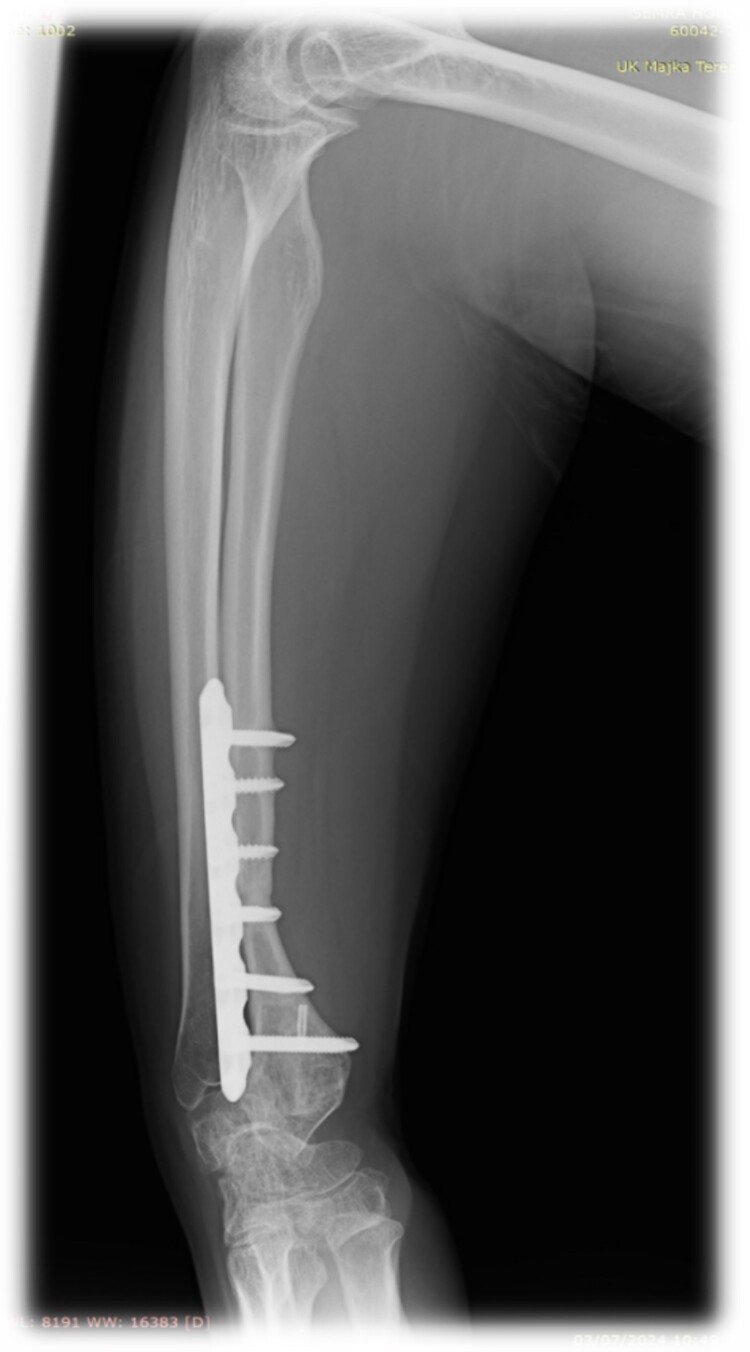
Follow-up X-ray of the right forearm 2 years after the surgery in profile plane

**Figure 8:**
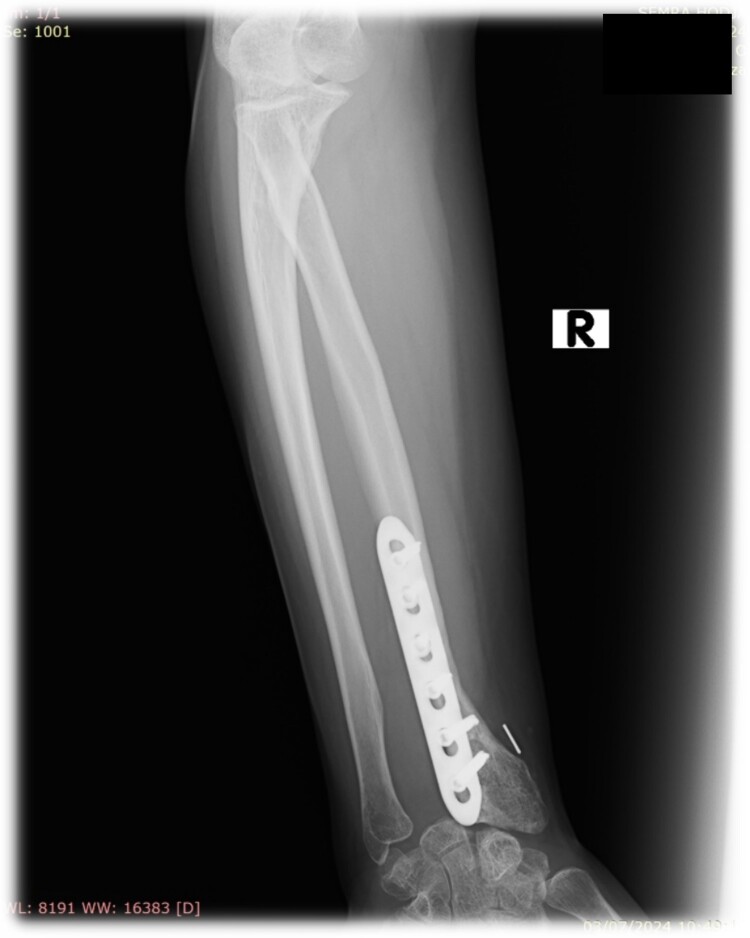
Follow-up X-ray of the right forearm 2 years after the surgery in anterior–posterior plane.

**Figure 9:**
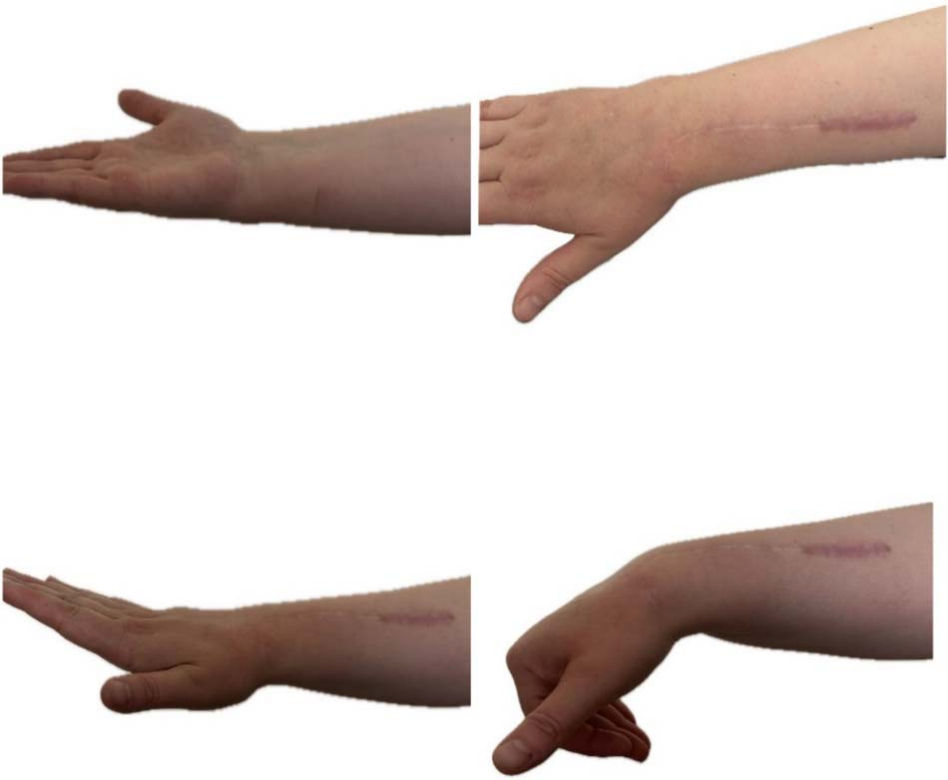
Range of motion in the wrist at 2-year follow-up.

At 2 years follow-up, no clinical and X-ray signs of tumor recurrence or graft resorption were detected, both X-ray and CT confirmed that bone healing has been achieved (Figs 7 and 8) On physical examination, the patient has decreased wrist dorsal and palmar flexion, all other wrist movements are in normal range of motion and without pain (Fig. 9).

## Discussion

Often preferred initial treatment for GCT of the distal radius is intralesional curettage with grafting to preserve wrist function, despite the high recurrence rates, associated with this technique [4].

After wide resection of the distal radius, many reconstruction procedures come in options such as arthroplasty, vascularized or non-vascularized fibular autograft with arthrodesis or without, allograft arthrodesis or osteoarticular allograft. These options have been more recently accepted by surgeons in terms of preserving the function and motion of the wrist [5].

Koul *et al.* in their study reports excellent and good functional results after reconstruction of the distal radius with vascularized fibular autograft, after wide resection of GCT [6].

Kocher *et al.* and Branchi *et al.* in their studies propose osteoarticular allograft reconstruction as an acceptable option for reconstruction, as a technique that provides good functional outcome and low recurrence rate [7, 8].

Vascularized and avascularized bone autografts have the same complications, but the advantages of using vascularized autografts are the live bone cells, the blood supply, and the ability for osteogenesis. The healing process is the biological advantage of these grafts, identical to fracture healing without an immune response. Another advantage is that the healing process is much shorter compared to nonvascularized autografts. Thus, according to Yunf-fa Yang, this graft may be the best replacement for the distal radius [9].

After detailed literature preview and taking into consideration all above-mentioned techniques and their advantages and disadvantages, in order to achieve local control of the tumor, we decided to perform reconstruction of the defect with free vascularized fibular autograft.

The nuclear medicine method—bone scintigraphy has been indicated as a very important diagnostic tool in monitoring vascularized bone grafts. Especially, the hybrid method of SPECT/CT is reliable in detecting the presence or absence of the radiotracer in the bone graft, predicting its failure. Studies suggest this tool is a reliable diagnostic method when an evaluation of graft viability is needed [10].

Moskowitz *et al.* described this method as unique due to the dependency of the tracer from both the delivery system and the viable network of osteocytes. He suggests that SPECT/CT can provide structural details of the graft and improved features. He describes this method as a simple, safe, and effective way to access both anastomosed vessels and the viability of the bone graft [11].

The presented surgical technique has encouraging results. It represents a safe technique for local control of the tumor and joint function preservation. As a limb salvage procedure, the autologous vascular fibular graft is an ideal substitute.

## References

[1] McCarthy EF . Giant-cell tumor of bone: an historical perspective. Clin Orthop 1980;153:14–25. 10.1097/00003086-198011000-00003. 7004712

[2] James SL , Davies AM. Giant-cell tumours of bone of the hand and wrist: a review of imaging findings and differential diagnoses. Eur Radiol 2005;15:1855–66. 10.1007/s00330-005-2762-5. 15868123

[3] Duncan CP , Morton KS, Arthur JF. Giant cell tumor of bone: its aggressiveness and potential for malignant change. Can J Surg 1983;26:475–6. 6351989

[4] Hess MC , Kafchinski L, Ransom E. Giant cell tumor of the distal radius: a review. Orthop Clin North Am 2023;54:75–88. 10.1016/j.ocl.2022.08.002. 36402513

[5] Duan H , Zhang B, Yang HS, et al. Functional outcome of en bloc resection and osteoarticular allograft reconstruction with locking compression plate for giant cell tumor of the distal radius. J Orthop Sci 2013;18:599–604. 10.1007/s00776-013-0394-1. 23661178

[6] Koul A , Patil R, Philip V, et al. Reconstruction of lower end of radius using vascularized upper end of fibula. Indian J Plast Surg 2007;40:61–6. 10.4103/0970-0358.32667.

[7] Kocher MS , Gebhardt MC, Mankin HJ. Reconstruction of the distal aspect of the radius with use of an osteoarticular allograft after excision of a skeletal tumor. J Bone Joint Surg Am 1998;80:407–19. 10.2106/00004623-199803000-00014. 9531209

[8] Bianchi G , Donati D, Staals EL, et al. Osteoarticular allograft reconstruction of the distal radius after bone tumour resection. J Hand Surg Br 2005;30:369–70. 10.1016/J.JHSB.2005.04.006. 15951074

[9] Yang YF , Wang JW, Huang P, et al. Distal radius reconstruction with vascularized proximal fibular autograft after en-bloc resection of recurrent giant cell tumor. BMC Musculoskelet Disord 2016;17:346. 10.1186/s12891-016-1211-8. 27530935 PMC4987985

[10] Kim H , Lee K, Ha S, et al. Predicting vascularized bone graft viability using 1-week postoperative bone SPECT/CT after maxillofacial reconstructive surgery. Nucl Med Mol Imaging 2020;54:292–8. 10.1007/s13139-020-00670-7. 33282000 PMC7704854

[11] Moskowitz GW , Lukash F. Evaluation of bone graft viability. Semin Nucl Med 1988;18:246–54. 10.1016/s0001-2998(88)80032-1 PMID: 3051396. 3051396

